# Aβ_42_ and ROS dual-targeted multifunctional nanocomposite for combination therapy of Alzheimer’s disease

**DOI:** 10.1186/s12951-024-02543-z

**Published:** 2024-05-23

**Authors:** Liding Zhang, Kai Cao, Jun Xie, Xiaohan Liang, Hui Gong, Qingming Luo, Haiming Luo

**Affiliations:** 1 State Key Laboratory of Digital Medical Engineering, Key Laboratory of Biomedical Engineering of Hainan Province, School of Biomedical Engineering, Hainan University, Haikou, 570228 China; 2 Britton Chance Center for Biomedical Photonics, Wuhan National Laboratory for Optoelectronics, MoE Key Laboratory for Biomedical Photonics, Huazhong University of Science and Technology, Wuhan, 430074 China; 3 Research Unit of Multimodal Cross Scale Neural Signal Detection and Imaging, Chinese Academy of Medical Sciences, HUST-Suzhou Institute for Brainsmatics, JITRI, Suzhou, 215123 China

**Keywords:** Alzheimer’s disease, Multifunctional nanocomposite, Aβ aggregation clearance, Reactive oxygen species, Tauopathy, Synergistic effect

## Abstract

Amyloid-β (Aβ) readily misfolds into neurotoxic aggregates, generating high levels of reactive oxygen species (ROS), leading to progressive oxidative damage and ultimately cell death. Therefore, simultaneous inhibition of Aβ aggregation and scavenging of ROS may be a promising therapeutic strategy to alleviate Alzheimer’s disease pathology. Based on the previously developed antibody 1F12 that targets all forms of Aβ_42_, we developed an Aβ_42_ and ROS dual-targeting nanocomposite using biodegradable mesoporous silica nanoparticles as carriers to load ultra-small cerium oxide nanocrystals (bMSNs@Ce-1F12). By modifying the brain-targeted rabies virus glycoprotein 29 (RVG29-bMSNs@Ce-1F12), this intelligent nanocomposite can efficiently target brain Aβ-rich regions. Combined with peripheral and central nervous system treatments, RVG29-bMSNs@Ce-1F12 can significantly alleviate AD symptoms by inhibiting Aβ_42_ misfolding, accelerating Aβ_42_ clearance, and scavenging ROS. Furthermore, this synergistic effect of ROS scavenging and Aβ clearance exhibited by this Aβ_42_ and ROS dual-targeted strategy also reduced the burden of hyperphosphorylated tau, alleviated glial cell activation, and ultimately improved cognitive function in APP/PS1 mice. Our findings indicate that RVG29-bMSNs@Ce-1F12 is a promising nanodrug that can facilitate multi-target treatment of AD.

**Supplementary Information:**

The online version contains supplementary material available at 10.1186/s12951-024-02543-z.

## Introduction

Alzheimer’s disease (AD) is an irreversible neurodegenerative disease characterized by progressive cognitive impairment and neuronal loss [1–3]. Abnormal accumulation of extracellular amyloid plaques in the brain is one of the main pathological features of AD. Amyloid-β (Aβ) peptide is a major component of senile plaques, among which Aβ_42_ is more prone to misfolding into different aggregates ranging in sizes from dimers to large fibrils [4, 5]. Although the exact role of Aβ_42_ aggregates in AD pathogenesis is unclear, most Aβ_42_ species are neurotoxic and lead to neuronal dysfunction and death [6–9]. Accumulating evidence indicates that Aβ aggregates are found in all regions of the brain in early AD [4, 10]. These neurotoxic aggregates generate high levels of reactive oxygen species (ROS) leading to progressive oxidative damage and ultimately causing cell death [11, 12]. Indeed, there is a good correlation between elevated Aβ load and elevated levels of oxidation products in the AD hippocampus and cortex, especially in Aβ-rich brain regions [13, 14]. In addition, excess ROS can accelerate tau phosphorylation and Aβ production, promoting the form of neurofibrillary tangles (NFTs) and plaques [15, 16]. Therefore, simultaneous inhibition of Aβ aggregation and scavenging excess ROS formation may be a promising therapeutic strategy to alleviate AD pathology.

Antibodies against Aβ_42_ aggregates have attracted considerable interest as potential therapeutic agents and research tools. Most anti-Aβ_42_ monoclonal antibodies (mabs), such as aducanumab (recognizing low molecular weight Aβ_42_ oligomer and fibrils) [17], lecanemab (recognizing Aβ_42_ protofibrils) [18], and gantenerumab (recognizing insoluble Aβ_42_) [19] preferentially target parts of Aβ aggregates rather than the monomer, suggesting that they recognize conformational epitopes. Although aducanumab and lecanemab have been approved by the FDA for AD treatment [17, 18], the clinical efficacy of these antibodies against the partial conformation of Aβ_42_ aggregates remains limited because the high level of Aβ_42_ monomers produced during pathological conditions may rapidly misfold into different aggregate forms. Therefore, passive immunotherapy targeting all forms of Aβ_42_ seems to be a promising treatment strategy. Our previously developed Aβ_42_ sequence- and conformation-specific antibody 1F12 can specifically bind to soluble Aβ_42_ monomers, oligomers, protofibrils, and insoluble fibers and plaque [20, 21]. Using this antibody against all forms of Aβ_42_ species may yield desirable therapeutic effects.

However, effective delivery of therapeutics to brain Aβ-rich regions remains a major challenge due to the blood-brain barrier (BBB) allows only approximately 0.1% of peripherally administered antibodies to enter the brain [22, 23]. Several strategies to enhance antibody uptake in brain have been developed through receptor-mediated transcytosis, of which rabies virus glycoprotein 29 (RVG29) is a promising brain-targeting peptide that primarily binds to nicotinic acetylcholine receptors (nAChR) and gamma-aminobutyric acid receptors (GABAAR) located on the surface of microvascular endothelial cells and neurons [24, 25]. Accordingly, we hypothesized that a safe biomaterial with RVG29 and anti-Aβ_42_ antibodies functionalized on its surface and loaded with ROS-scavenging agents could inhibit Aβ aggregation, accelerate Aβ_42_ clearance, and scavenge ROS. We believed that biodegradable mesoporous silica nanoparticles (bMSNs), with their biodegradable nature and high loading efficiency, would be the ideal model material to test this hypothesis [26, 27].

**Fig. 1:**
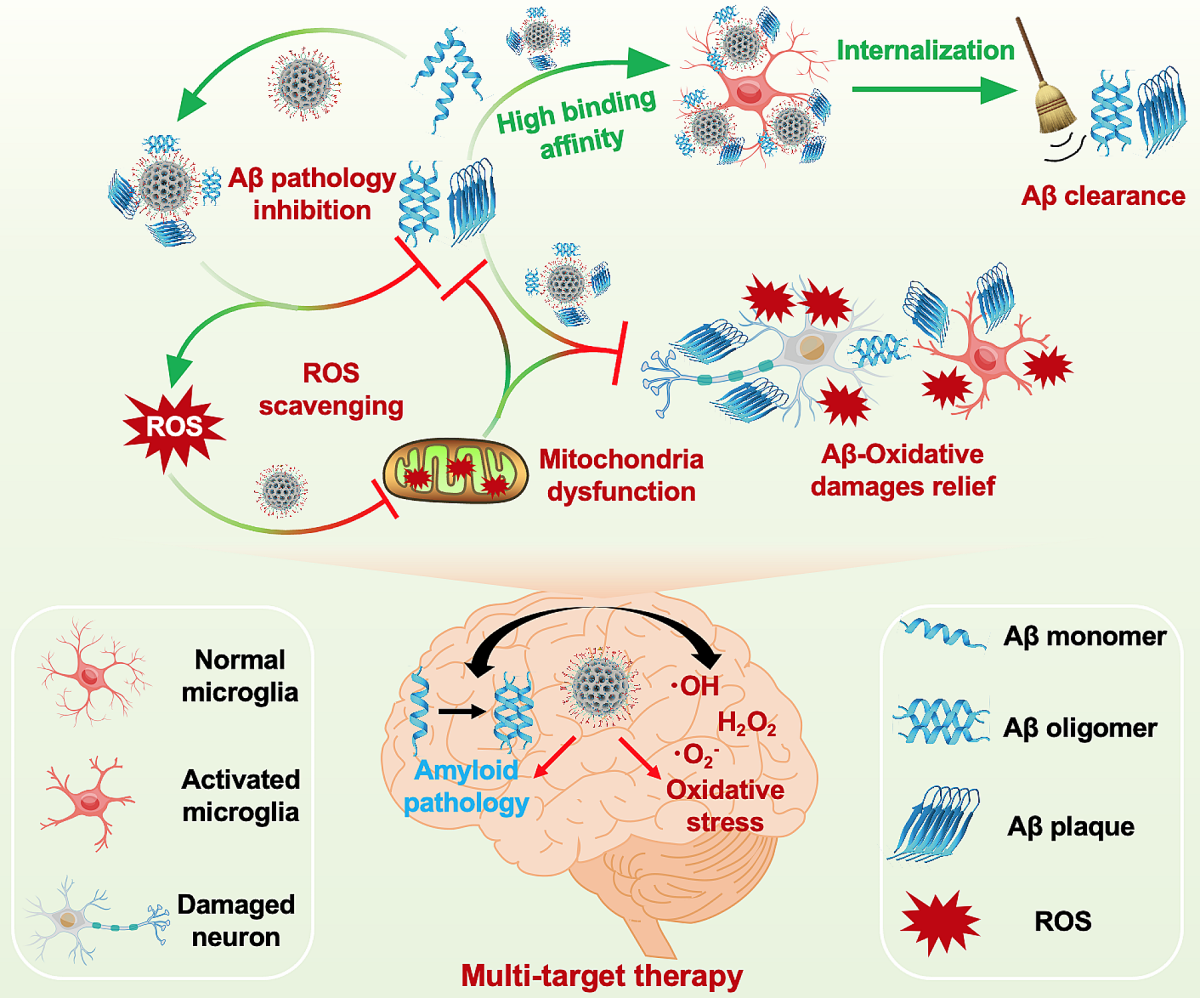
Illustration of RVG29-bMSNs@Ce-1F12 for combinational therapy of AD

bMSNs are relatively safe biomaterials characterized by high porosity, tunable pore size, high specific surface area, and easy functional modification of the surface [28, 29]. Further, the presence of two different oxidation states of cerium (Ce^3+^ and Ce^4+^) of cerium oxide nanoparticles (CeNPs) can mimic a series of natural redox enzymes, including superoxide dismutase (SOD)_3_ and catalase, to scavenge harmful ROS from the body [30, 31]. The enzymatic activity of CeNPs for scavenging ROS is a result of the self-regeneration cycle of Ce^3+^/Ce^4+^ and the oxygen vacancies on the cerium surface [32]. Thus, bMSNs can scavenge ROS from the brain by loading CeNPs to reduce ROS-induced neuronal apoptosis, tau phosphorylation, and Aβ production, thereby inhibiting their aggregation and eventually the formation of NFTs and plaques. In addition, bMSNs can rapidly be excreted from the liver into the gastrointestinal tract [33]. In this study, bMSNs were loaded with CeNPs and chemically functionalized with anti-Aβ_42_ antibody 1F12 and RVG29 to bind simultaneously to Aβ_42_ and scavenge ROS (Fig. 1). The specific clearance of pathogenic Aβ_42_ and ROS scavenging enables the reduction of Aβ and ROS levels and attenuates a cascade of downstream damages induced by the two. The creative concept and biotechnological approach presented here open an avenue to delay the progression of AD by eliminating brain-derived Aβ_42_ and ROS in the peripheral and central nervous system (CNS).

## Materials and methods

### Materials

Aβ_42_ was custom-synthesized as a lyophilized powder by Royo Biotech Co., Ltd (Shanghai, China) with > 95% purity. Monoclonal antibody 1F12, subtype IgG2a, recognizes the 3–9 amino acid located in the N-terminal region of the Aβ_42_ peptide and selectively binds all forms of Aβ_42_ [20, 21]. MonoRab™ anti-mouse IgG (H + L) and protein A resin were ordered from GenScript (Nanjing, China). Bovine serum albumin (BSA), 3-aminopropyl triethoxysilane, and Tris were purchased from Sigma-Aldrich. Cy3-NHS ester was provided by Yeasen Biotechnology Co., Ltd (Shanghai, China). SOD assay kit-WST was ordered from Tongren Institute of Chemistry (Dorento, Japan). Cetyltrimethylammonium chloride (CTAC), triethanolamine (TEA), chloroform, tetraethyl orthosilicate (TEOS), cyclohexane, Phenyl ether, and N, N-Dimethylformamide (DMF) were supplied from Sinopharm Chemical Reagent Co., Ltd. (Shanghai, China). N-hydroxysuccinimide (NHS), 1-Ethyl-3-(3-dimethylaminopropyl) carbodiimide hydrochloride (EDC-HCl), oleic acid, and oleylamine were obtained from Aladdin (Shanghai, China). Citric acid was obtained from Innochem (Beijing, China). Bromo-2-methylpropionic acid (BMPA), oleyl alcohol, and cerium acetate were purchased from Macklin Inc. (Shanghai, China). Dimethylbenzene was provided by Kermel (Tianjin, China). 2′,7′-dichlorofluorescein diacetate (DCFH-DA) was supplied from Servicebio (Wuhan, China).

### Synthesis of amine-modified biodegradable mesoporous silica nanoparticles (bMSNs)

bMSNs were synthesized according to a previously reported protocol [34, 35]. Briefly, 24 mL of CTAC solution (25 wt%) was first heated to 60 °C for 5 min under gentle agitation. Subsequently, TEA (0.18 g) dissolved in 2 mL of deionized water was added to the CTAC solution and kept at 60 °C for another 1 h. Then 20 mL of TEOS (20 v/v%, cyclohexane) was slowly dripped and stirred gently at 60 °C for 20 h. After cooling to room temperature, the upper organic phase was removed. The pellet was collected by centrifugation at 10,000 rpm for 10 min and washed three times with ethanol. The product was then dissolved in 50 mL of NaCl-ethanol (1 w/v%) and stirred at room temperature for 24 h to remove the template. Finally, the product was collected by centrifugation and dispersed in ethanol for further use.

To synthesize amine-modified bMSNs (NH_2_-bMSNs), 100 mg of bMSNs were dissolved in 100 mL of ethanol and heated to 88 °C for 10 min. Then, 1 mL of APTES was added and the reaction was stirred at 88 °C for 48 h. The prepared NH_2_-bMSNs were collected by centrifugation and then dispersed in DMF.

### Synthesis of ceria nanoparticles (CeNPs)

Cerium (III) acetate (0.43 g) and oleylamine (3.25 g) were dissolved in 15 mL of dimethylbenzene and stirred at room temperature for 24 h. The mixture was gradually heated to 90 °C under an Ar atmosphere, and then 1 mL of deionized water was quickly added and kept at 90 °C for 3 h. Then, the mixture was rapidly cooled to room temperature and the product was pelleted by adding 100 mL of acetone followed by centrifugation. The resulting precipitate (15 mg) was dispersed in a mixture of chloroform and DMF (50/50 v/v, 30 mL), and then BMPA (0.5 g) and citric acid (0.05 g) were added to react for another 3 h at room temperature to obtain BMAP-capped CeNPs [34].

### Synthesis of bMSNs@Ce

To synthesize bMSNs@Ce, NH_2_-bMSNs (10 mg) and CeNPs (5 mg) were dissolved in DMF (10 mL). After stirring at room temperature for 12 h, a precipitate was obtained and excess CeNPs were removed by washing with DMF. The synthesized bMSNs@Ce was lyophilized in DMSO for further use.

### Synthesis of bMSNs@Ce modified with RVG29 and 1F12

bMSNs@Ce with RVG29 and 1F12 modification were obtained by the following steps. First, 10 mg of bMSNs@Ce were resuspended in 300 µL of MES buffer (100 mM MES, pH 5.0) and then 100 µL of freshly prepared EDC (50 mg/mL) and NHS (50 mg/mL) were added to homogenize by ultrasound. Then, 50 µg of RVG29 and 300 µg of anti-Aβ_42_ 1F12 were added to react with shaking for 4 h. Afterward, RVG29-bMSNs@Ce-1F12 was collected by centrifugation and washed twice with PBS. The synthesized RVG29-bMSNs@Ce-1F12 were stored at 4 °C until use.

### Characterization of the prepared NPs

The morphology of the synthesized bMSNs, CeNP, bMSNs@Ce, and RVG29-bMSNs@Ce-1F12 was characterized by Tecnai G20 (FEI Ltd., NL), Nova Nano scanning electron microscopy (SEM) (FEI., NL) and SPM9700 atomic force microscope (AFM) (Shimadzu., Japan). Dynamic light scattering and Zeta potential were performed on a Zetasizer Nano ZS90 device (Malvern Instruments, UK). XRD was scanned by x’pert3 powder (PANalytical B.V., Holland). The element types and contents of bMSNs@Ce were analyzed by EDS (Sirion 200, EFI, Holland). The valence states of elements were analyzed by XPS (AXIS-ULTRA DLD-600 W, Kratos, Japan). Fourier transform infrared spectrometer (FT-IR) was scanned by Nicolet iS50R (Thermo Scientific, USA).

### Superoxide dismutase mimetic activity assay

The superoxide anion scavenging activity of RVG29-bMSNs@Ce-1F12 was measured using a superoxide dismutase (SOD) assay kit-WST (Dorento, Japan) according to the manufacturer’s instructions. Briefly, RVG29-bMSNs@Ce-1F12 dispersions with different Ce element concentrations (including 0, 0.0625, 0.125, 0.25, 0.5, and 1 mM) were mixed with 200 µL of WST® working solution and added to each well. Next, 20 µL of xanthine oxidase solution was added to start the reaction by incubation at 37 °C for 20 min, and then the absorbance at 450 nm was measured using an Epoch microplate spectrophotometer (Bio Tek, USA). The SOD-mimetic activity of RVG29-bMSNs@Ce-1F12 dispersions with different concentrations of the Ce can be measured by quantifying the absorbance at 450 nm, since the absorbance is proportional to the amount of superoxide anion.

#### Hydroxyl radical scavenging ability assay

The hydroxyl radical scavenging ability of RVG29-bMSNs@Ce-1F12 was measured using a hydroxyl free radical scavenging ability test kit (Solarbio, China) according to the manufacturer’s instructions. Briefly, RVG29-bMSNs@Ce-1F12 dispersions with different Ce element concentrations (including 0, 0.0625, 0.125, 0.25, 0.5, and 1 mM) were mixed with working buffer. The hydroxyl radical scavenging activity of RVG29-bMSNs@Ce-1F12 dispersions with different concentrations of the Ce can be measured by quantifying the absorbance at 536 nm.

### Cell culture and cell proliferation assays

SH-SY5Y and BV2 cell lines were cultured in DMEM/F12 medium (Gibico, USA) containing 10% fetal bovine serum (FBS, Vazyme Biotech Co., Ltd, Nanjing, China), and hybridoma cells (1F12) were cultured in RMPI-1640 medium (Gibico, USA) containing 20% FBS in 5% CO_2_ at 37 °C. Cell viability was measured using a CellTiter 96 Aqueous One Solution Cell Proliferation Assay kit (Promega, USA) according to the manufacturer’s instructions. SH-SY5Y or BV2 cells (1 × 10^4^/well) were seeded in 96-well plates (NEST Biotechnology Co., Ltd, Wuxi, China) for 24 h and then treated with different concentrations of RVG29-bMSNs@Ce-1F12 dispersion from 10 to 100 µg/mL for 12 h to study biocompatibility. Untreated SH-SY5Y cells or BV2 cells were used as controls. Since absorbance is proportional to cell proliferation, cell viability was measured by quantifying the value at 490 nm.

### ThT assay

Aβ_42_ monomers and aggregates were prepared as previously described [21]. In the presence of PBS, RVG29-bMSNs@Ce, 1F12, or RVG29-bMSNs@Ce-1F12, the process of Aβ_42_ aggregation was dynamically monitored by ThT, a fluorescent probe binds specifically to β sheet-rich structures and enhances fluorescence intensity [36]. Briefly, aggregated Aβ_42_ in each treatment group was diluted to 20 µM and mixed with 50 µM of ThT. Then, the fluorescence spectrum and intensity of the samples were detected by a multi-mode microplate reader (FlexStation3; Molecular Devices, USA) under 440 nm excitation and 480 nm emission.

### Inhibition of Aβ oligomerization and aggregation in vitro

Aβ_42_ monomers were incubated with RVG29-bMSNs@Ce, 1F12, or RVG29-bMSNs@Ce-1F12, respectively, while PBS-treated Aβ_42_ was used as a control. Samples were incubated at 37 °C for 24 h and then centrifuged at 12,000 g for 30 min to obtain soluble Aβ_42_. The extent of oligomerization of Aβ_42_ was determined by sandwich enzyme-linked immunosorbent assay (ELISA) and ThT assay. The morphology of Aβ_42_ aggregates in each treatment group was observed by TEM.

### Evaluation of the ability of RVG29-bMSNs@Ce-1F12 to depolymerize Aβ_42_ protofibril

Prepared Aβ_42_ protofibril containing Aβ_42_M and low molecular weight Aβ_42_ oligomers were dialyzed in PBS solution containing RVG29-bMSNs@Ce-1F12 (50 µg/mL) using 14 kDa molecular weight cutoff dialysis bags. The dialysate was changed every 4 h for 48 h. The effect of RVG29-bMSNs@Ce-1F12 on the acceleration of Aβ_42_ fibrils depolymerization was evaluated by ELISA, ThT, and TEM.

### Dtection of ROS levels

For the detection of ROS levels in cells, SH-SY5Y and BV2 cells at a concentration of 1 × 10^5^ cells/mL were seeded in 6-well plates (NEST Biotechnology Co., Ltd, Wuxi, China) and cultured at 37 °C, 5% CO_2_ for 12 h. Cells were then co-incubated with 10 µM of Aβ_42_ pretreated with PBS, RVG29-bMSNs@Ce, 1F12, or RVG29-bMSNs@Ce-1F12 at 37 °C, 5% CO_2_ for 1 h. After incubation, cells from each treatment group were washed with PBS and then incubated with 2′,7′-dichlorofluorescin diacetate (DCFH-DA, Servicebio, Wuhan, China) for 30 min at 37 °C in a 5% CO_2_ incubator. After washing with PBS, the ROS fluorescence signal of cells were observed using a Zeiss LSM710 confocal laser microscope.

For the detection of ROS levels in plasma and brain tissues, the plasma samples and brain tissue supernatant collected from each treatment group were mixed with TBS buffer and then incubated with DCFH-DA (50 µM) at 37 °C for 30 min. Afterwards, a multi-mode microplate reader was used to quantitatively detect the fluorescent signals of all samples with an excitation wavelength of 488 nm and an emission wavelength of 525 nm.

### Cellular uptake of Aβ_42_

To assess the cellular uptake of Aβ_42_ in each treatment group, Cy3-labeled Aβ_42_ (50 µM) was incubated with PBS, RVG29-bMSNs@Ce, 1F12, or RVG29-bMSNs@Ce-1F12 at 37 °C for 12 h. BV2 cells were then incubated with 10 µL of the above-prepared solution containing 5 µM Aβ_42_ at 37 °C for 12 h. Afterwards, cells from each group were washed with PBS, and fluorescence signals were detected by confocal laser microscopy.

After incubation, the total protein of BV2 cells was extracted using RAPI buffer (Solarbio, Beijing, China) and then quantified by NanoDrop One spectrophotometer (Thermo Fisher). The levels of Aβ_42_ uptake by BV2 cells was quantitatively by sandwich ELISA.

### Animal

APP/PS1 transgenic mice (Stock No: 034832-JAX) and C57BL/6J mice were ordered from Jackson Laboratory and Liaoning Changsheng biotechnology Co., Ltd, respectively. All procedures related to animal research have been reviewed and approved by the Institutional Animal Care and Use Committee of Huazhong University of Science and Technology.

### In vivo tracking of nanocomposites in mice

Cy3-labeled RVG29-bMSNs@Ce, 1F12, or RVG29-bMSNs@Ce-1F12 were all intravenously administered into 2-month-old C57BL/6J mice, and whole-body fluorescence imaging was performed to monitor their dynamic biodistributions at 0.5, 1, 2, 4, and 6 h with filter set (excitation = 562/40 nm; emission = 775/46 nm). Mice were sacrificed after imaging, and 15 μm coronal cryosections of liver and intestine were stained with 4’,6-diamidino-2-phenylindole (DAPI) for 10 min at room temperature and imaged using a confocal laser microscope.

### BBB permeability in vivo

bEnd.3 (6 × 10^4^ cells/transwell) was cultured in 12 wells for 7 days to form a compact monolayer to mimic the BBB. On the 5th day of culture, SH-SY5Y cells were seeded into the bottom of 12 wells. To evaluate the permeability across the monolayer, Cy3-labeled 1F12, RVG29-bMSNs@Ce, or RVG29-bMSNs@Ce-1F12 dissolved in PBS was added to the culture medium and incubated apically for 4 h. Afterwards, the fluorescence of SH-SY5Y was detected by confocal laser microscopy.

### Evaluation of the effect of RVG29-bMSNs@Ce-1F12 brain targeting and Aβ plaques labeling

To evaluate the performance of RVG29-bMSNs@Ce-1F12 brain targeting and labeling Aβ plaques in the brains of APP/PS1 mice, 14-month-old APP/PS1 mice were injected intravenously with Cy3-labeled RVG29-bMSNs@Ce-1F12 at a dose of 10 mg/kg. After 4 h of post-injection, mice were anesthetized and brain fluorescence was detected by whole-body fluorescence imaging. Mice were sacrificed after imaging and brain slices were prepared and stained with thioflavin S to evaluate the effect of RVG29-bMSNs@Ce-1F12 on Aβ plaque labeling.

### In vivo degradation of bMSNs@Ce

For the biodegradation test, bMSNs@Ce NPs were dispersed in lactated Ringer’s solution to a final concentration of 1 mg/mL. The mixture was incubated at 37 °C for 7 days. The resulting product was centrifuged at 10,000 rpm for 10 min and resuspended in ethanol. The degradation degree of bMSNs@Ce was observed by TEM.

### ELISA assays

For indirect ELISA, Aβ_42_ was coated in wells of 96-plates overnight at 4 °C. After blocking with 5% milk, 1F12 or RVG29-bMSNs@Ce-1F12 was added to the wells for 2 h incubation at 37 °C, followed by incubation with horseradish peroxidase (HRP)-conjugated goat anti-mouse IgG (H + L) (1:8000, GenScript, Nanjing, China). After each step, 96 plates were washed 3 times with phosphate-buffered saline containing 0.05% Tween-20 (PBS-T). Immunoreactive signals were detected by incubation with TMB substrate solution (Abcam) for 15 min at 37 °C and then measured by an Epoch Microplate Spectrophotometer at 450 nm.

Plasma or tissues from APP/PS1 mice were extracted with a tissue grinder in tris-buffered saline containing complete protease inhibitor cocktail (Roche) at a ratio of 1:50 (w/v). The homogenate supernatant was centrifuged at 12,000 g for 30 min at 4 °C to obtain TBS-soluble protein. Aβ_42_ and p-tau^396,404^ levels in soluble proteins were quantified by our previously prepared sandwich ELISA [21, 37].

### Immunoprecipitation and Western blot

Brain homogenate or plasma was incubated with 40 µg/mL of 2C6-conjugated protein A/G magnetic beads (LinkedIn Biotechnology Co., Ltd., Shanghai, China) for 30 min at room temperature according to the manufacturer’s instructions. Immunoprecipitated proteins were eluted with 0.1 M glycine (pH 3.0) and immediately neutralized to pH 7.4 with neutralization buffer (1 M Tris-HCl, pH 8.5). Samples were denatured by boiling for 10 min in loading buffer (Booster Biotech, Shanghai, China), and proteins were then run in 12% SDS-PAGE. Proteins were transferred to polyvinylidene fluoride membranes at 160 mA for 1 h. Membranes were blocked with 5% skimmed milk and incubated with 2C6 (1:2000) at 37 °C for 2 h. Membranes were then washed with PBS-T, followed by 1 h incubation with HRP-conjugated goat anti-mouse IgG (H + L) antibody (1:8000). The immune signals were visualized with ECL-substrate (Yeasen Biotechnology Co., Ltd, Shanghai, China) and detected with a Tanon 5200 Muiti (Shanghai, China).

### Immunofluorescence staining

Mouse brain tissues from different treatment groups were collected, post-fixed in 4% PFA, and dehydrated in sucrose solution. A series of 15 μm coronal cryosections of the above tissue samples were collected and permeabilized with 0.2% Triton X-100 for 20 min at room temperature. Tissue sections were then blocked with 3% BSA for 2 h at room temperature and incubated with Cy3-labeled anti-Aβ_42_ mouse mAb 2C6 (1:1000) [20, 21], anti-p-tau^396,404^ mouse mAb 4B1 (1:600) [37], or Iba1/AIF-1 (E4O4W) XP® rabbit mAb (1:800) overnight at 4 °C. All slides were washed five times with TBS and stained with DAPI or thioflavin S. Fluorescent signals were detected using a Zeiss LSM710 microscope.

### Olfactory behavior testing and nesting

Mice were screened for olfactory deficits using the odor cross-habituation test as previously described [38]. Odors (*n* = 6; limonene, ethyl valerate, isoamyl acetate, pentanol, heptanone, and nonane) were diluted in castor oil for olfactory behavior testing. The duration of the investigation was defined as nose-oriented sniffing within 1 cm of the odor presentation port. In the nesting test, mice in each treatment group were divided into single cages and adapted to the single-cage environment for 48 h. Then, sheets of paper (5 × 5 cm^2^) were placed in the cage to provide conditions for mice to build their nests. Nest specific qualities were based on previously described criteria [39, 40].

#### Morris water maze

The Morris water maze test was used to evaluate the spatial memory function of APP/PS1 mice after treatment. Before training, the pool was filled with water kept at 24–26 °C and stained with white ink. During the first five days, the mice in each treatment group were placed into the water pool from different quadrants, and they swam freely to find the hidden platform. Swim speed, swim path, and latency to find the platform were recorded. During each training session, the mice were given 60 s to find the platform. If the mice did not find the platform, they were helped to find it and allowed to stay on the platform for 15 s. The training sessions were repeated 4 times per day for five days. Finally, on the sixth day, the space exploration test was conducted by removing the hidden platform and placing the mice into the pool from the opposite side of the original platform. Their behavior within 60 s was recorded by camera and analyzed by Visutrack animal behavior analysis software (Shanghai Xinsoft Information Technology Co., Ltd).

### Statistical analysis

Data are presented as mean ± SD. An unpaired t-test was used for comparisons of two groups. One-way analysis of variance (ANOVA) was used for multiple group comparisons. Statistical significance is present in the Figure by ^*^p < 0.05, ^**^p < 0.01, ^***^p < 0.001, ^****^p < 0.0001, and ns (indicates no significance). All statistical analyzes were performed with GraphPad Prism 8.0 software.

## Results and discussion

### Synthesis and characterization of RVG29-bMSNs@Ce-1F12

**Fig. 2:**
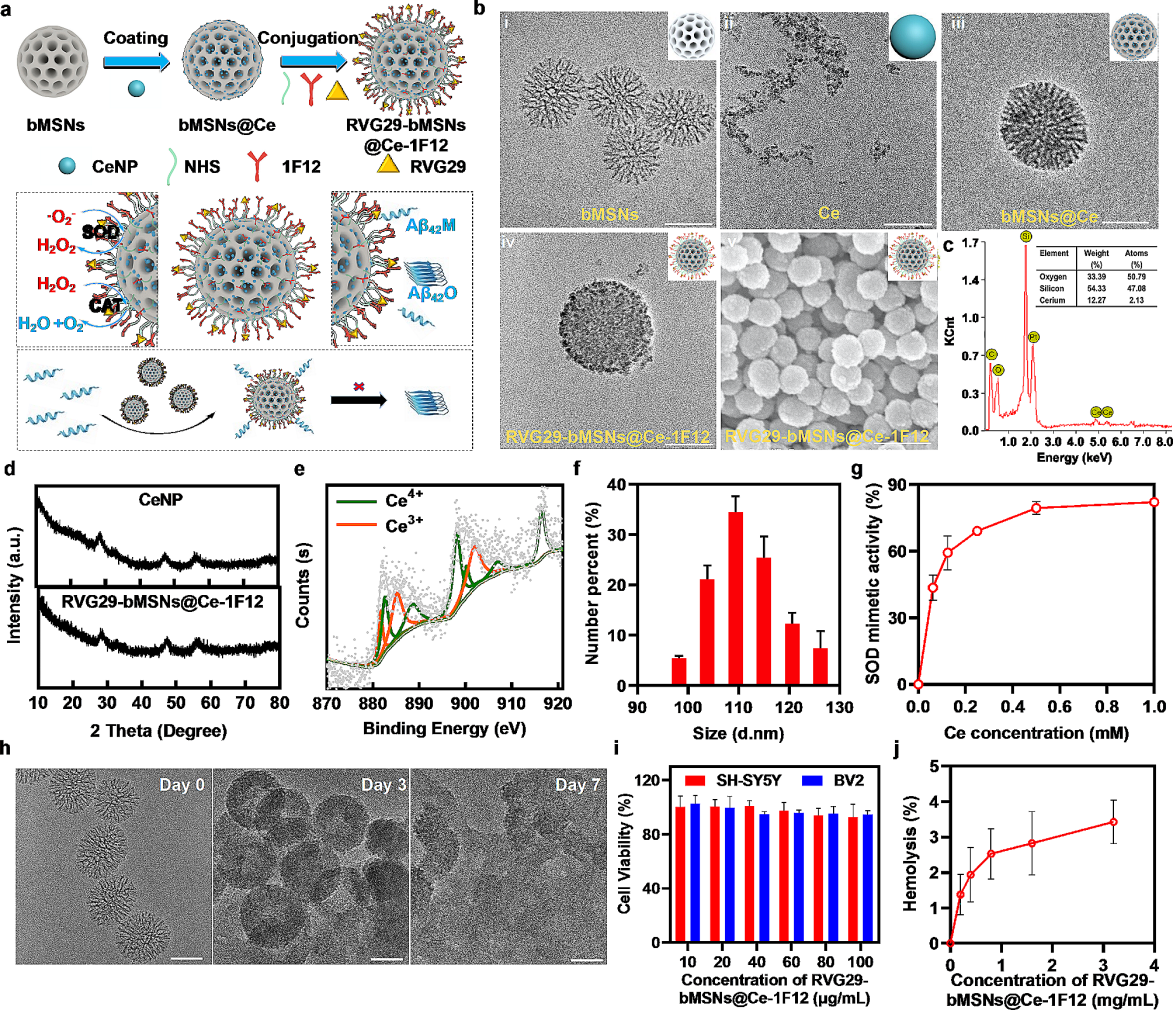
Characterization of RVG29-bMSNs@Ce-1F12. (**a**) Schematic illustration of RVG29-bMSNs@Ce-1F12 synthesis process and its therapeutic mechanism. (**b**) TEM images of bMSNs (i), CeNPs (ii), bMSNs@Ce (iii), and RVG29-bMSNs@Ce-1F12 (iv); SEM image of RVG29-bMSNs@Ce-1F12 (v). Scale bar = 50 nm for i-iv, scale bar = 200 nm for v. (**c**) EDS analysis (left) and quantification of different elements (right) of bMSNs@Ce. (**d**) XRD patterns of CeNPs and RVG29-bMSNs@Ce-1F12. (**e**) XPS analysis of RVG29-bMSNs@Ce-1F12. (**f**) Hydrodynamic diameter of RVG29-bMSNs@Ce-1F12. (**g**) Neutralization of superoxide anions by RVG29-bMSNs@Ce-1F12 in a dose-dependent manner. (**h**) TEM images of bMSNs@Ce degradation process. Scale bar = 50 nm. (**i**) Dose-dependent cytotoxicity of RVG29-bMSNs@Ce-1F12 on SH-SY5Y and BV2 cells. (**j**) Statistical results of hemolysis rate under different concentrations of RVG29-bMSNs@Ce-1F12. Data are presented as mean ± SD, *n* = 3

Smart multifunctional RVG29-bMSNs@Ce-1F12 was constructed by anchoring CeNPs to bMSNs, followed by surface modification with RVG29 and 1F12 for brain and Aβ_42_ targeting. Figure 2a summarizes the fabrication process of RVG29-bMSNs@Ce-1F12 and its underlying therapeutic mechanism. The surface of bMSNs anchoring CeNPs was modified with RVG29 and 1F12 (Fig. S1). The size and Zeta potential of synthetic amino modified bMSNs were approximately 60 nm and 34.7 mV (Fig. 2b(i), Fig. S2a, and Fig. S2d). The modified CeNPs immobilized on the surface of bMSNs were highly crystallized, with a size of approximately 2.7 nm (Fig. 2b(ii) and Fig. S2b). The results of TEM, energy dispersive X-ray spectroscopy (EDS) mapping, and X-ray diffraction (XRD) (Fig. 2b(iii) and Fig. 2c) confirmed the successful immobilization of CeNPs on bMSNs, with the size of bMSNs@ce was approximately 91 nm (Fig. S2c). X-ray photoelectron spectroscopy (XPS) results showed that RVG29-bMSNs@Ce-1F12 had mixed valency states for CeNPs, and their corresponding-binding energy peaked at 901.8, 885.1, and 881.6 eV for Ce^3+^ and at 916.5, 906.8, 900.1, 898.1, 888.4, and 882.5 eV for Ce^4+^ (Fig. 2d, e, and Fig. S3). The proportion of Ce^3+^ in RVG29-bMSNs@Ce-1F12 was 42.53%, indicating that RVG29-bMSNs@Ce-1F12 could act as a ROS scavenger because of its transition between Ce^3+^ and Ce^4+^. TEM, AFM, and SEM images showed that RVG29-bMSNs@Ce-1F12 was uniform and well-dispersed in water (Fig. 2b(iv, v), and Fig. S4), with a hydrodynamic size of approximately 110 nm and Zeta potential of 56.1 mV (Fig. 2f, and Fig. S2d). Enzyme-linked immunosorbent assay (ELISA) and sodium dodecyl sulfate-polyacrylamide gel electrophoresis (SDS-PAGE) confirmed the successful modification of RVG29 and 1F12 on the surface of bMSNs@Ce. ELISA showed positive signals of RVG29 in the sample of RVG29-bMSNs@Ce-1F12 using an anti-his tag, and positive signals of 1F12 using an anti-mouse IgG antibody (Fig. S5a). SDS-PAGE images showed 1F12 and RVG29 protein bands in the lane with RVG29-bMSNs@Ce-1F12 (Fig. S5b). Furthermore, the SOD simulation test and hydroxyl radical scavenging ability confirmed that RVG29-bMSNs@Ce-1F12 maintained the antioxidative property of CeNPs (Fig. 2g and Fig. S6).

### Biocompatibility of RVG29-bMSNs@Ce-1F12 in vitro and in vivo

Nanomedicines used for AD treatment should have minimal toxicity and good biocompatibility [41]. To evaluate the degradation ability of bMSNs@Ce, bMSNs@Ce was incubated with lactated Ringer’s solution for different durations. The results showed that bMSNs@Ce was uniformly dispersed before degradation but became shapeless after 7 days of incubation, indicating the gradual degradation of bMSNs@Ce (Fig. 2h). The results of cell viability analysis showed that RVG29-bMSNs@Ce-1F12 showed no obvious toxicity to SH-SY5Y and BV2 cells at a concentration of 100 µg/mL (Fig. 2i). Moreover, no significant change was observed in the cell morphology of SH-SY5Y and BV2 cells after exposure to RVG29-bMSNs@Ce-1F12 (Fig. S7a). Besides, hemolysis experiments were performed to verify the biocompatibility of the material. The results showed that when the concentration of RVG29-bMSNs@Ce-1F12 was 3.2 mg/mL, its hemolysis rate was less than 3.5%, which was 16 times higher than the injection dose (Fig. 2j and Fig. S7b). According to ISO 10993-4 [42], RVG29-bMSNs@Ce-1F12 can be considered as a feasible blood-contacting material with a hemolysis rate of less than 5%.

Considering the high liver accumulation of RVG29-bMSNs@Ce-1F12 resulting from an intravenous injection, the toxicity of RVG29-bMSNs@Ce-1F12 was further investigated in vivo via a histopathological assay (Fig. S7c). Hematoxylin-eosin-stained images of the heart, liver, spleen, lung, and kidney showed a highly similar morphology between saline-treated mouse samples and RVG29-bMSNs@Ce-1F12-treated samples. The abovementioned results indicate that RVG29-bMSNs@Ce-1F12 has low toxicity and high biocompatibility.

#### Aβ_42_ targeting ability of RVG29-bMSNs@Ce-1F12

**Fig. 3:**
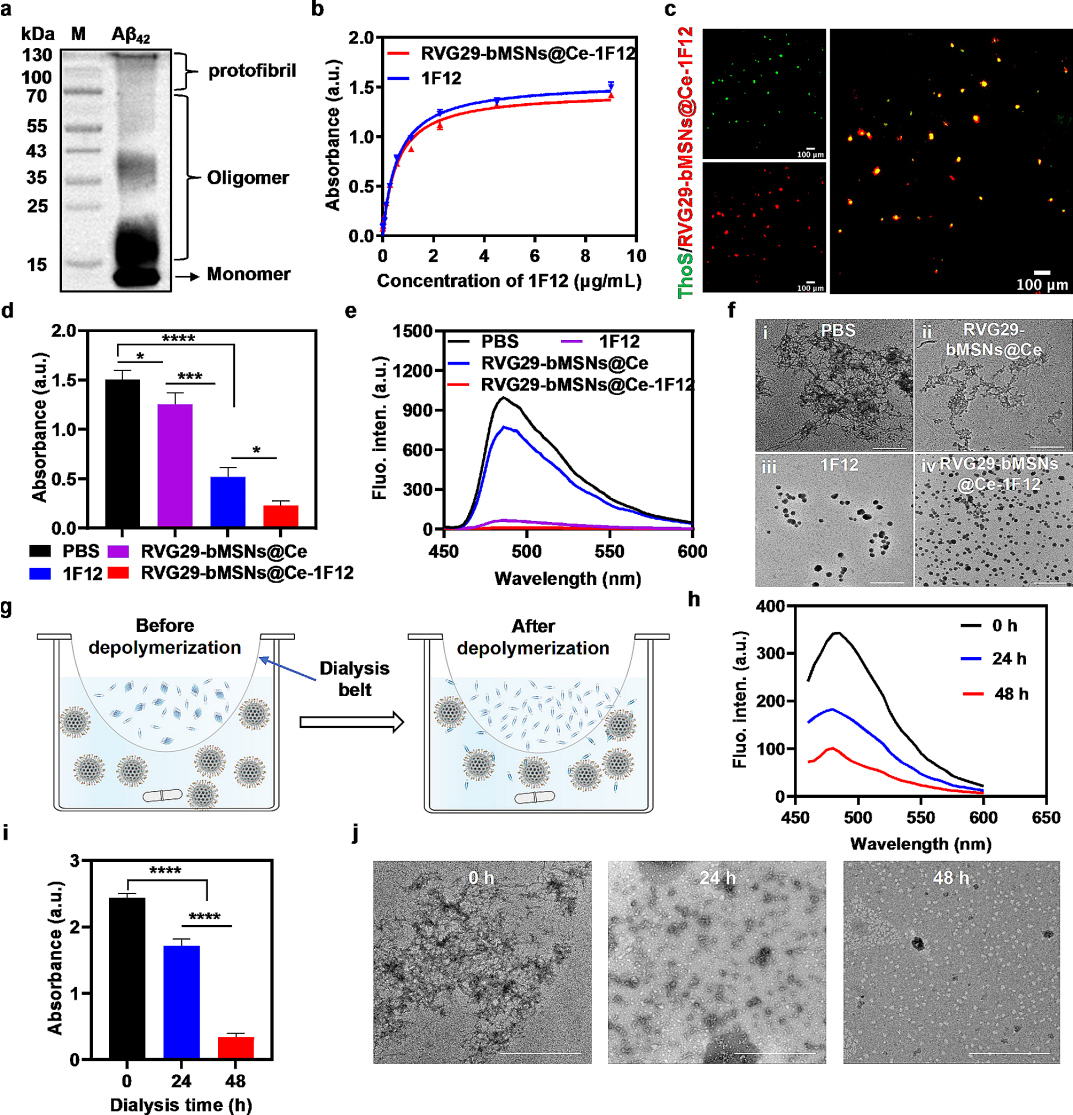
Amyloidogenesis inhibition by RVG29-bMSNs@Ce-1F12 in vitro. (a) Different forms of Aβ_42_ are identified via IP-Western blot analysis of RVG29-bMSNs@Ce-1F12. (b) The binding affinity of RVG29-bMSNs@Ce-1F12 toward Aβ_42_. (c) Confocal images of Aβ plaques stained with RVG29-bMSNs@Ce-1F12 (red) and thioflavin S (green). Scale bar = 100 μm. (d-f) ELISA, ThT, and TEM assessment of Aβ_42_ aggregation in the presence of PBS, RVG29-bMSNs@Ce, 1F12, and RVG29-bMSNs@Ce-1F12. (g) Schematic illustration of the depolymerization of Aβ_42_ profibrils in the presence of RVG29-bMSNs@Ce-1F12. (h-j) ThT, ELISA, and TEM assessment of the depolymerization of Aβ_42_ fibrils in the presence of RVG29-bMSNs@Ce-1F12. Data are presented as mean ± SD, *n* = 3. One-way analysis of variance (ANOVA) was performed for multigroup comparisons, *p < 0.05, ***p < 0.001, and ****p < 0.0001

We first assessed the ability of RVG29-bMSNs@Ce-1F12 to target Aβ_42_ monomers, oligomers, and protofibrils. Aβ_42_ species were enriched by RVG29-bMSNs@Ce-1F12-based immunoprecipitation followed by Western blot (IP-Western blot); the results showed that RVG29-bMSNs@Ce-1F12 inherited the targeting ability of 1F12 and could specifically recognize soluble Aβ_42_ monomers, oligomers, and protofibrils (Fig. 3a). The affinity test results showed that 1F12 (*K*_*d*_ = 3.79 ± 0.226 nM) and RVG29-bMSNs@Ce-1F12 (*K*_*d*_ = 3.95 ± 0.256 nM) had similar *K*_*d*_ values toward Aβ_42_, indicating the high binding affinity of RVG29-bMSNs@Ce-1F12 toward Aβ_42_ (Fig. 3b). Subsequently, the insoluble Aβ plaque targeting ability was evaluated using Cy3-labeled RVG29-bMSNs@Ce-1F12. Confocal imaging showed Aβ plaques were labeled by RVG29-bMSNs@Ce-1F12-Cy3 and co-localized with thioflavin S (Fig. 3c). The labeling effect observed with RVG29-bMSNs@Ce-1F12 was comparable to that observed for 1F12 (Fig. S8a). However, for RVG29-bMSNs@Ce, no colocalized fluorescence signal was observed (Fig. S8b). Taken together, the results indicate that RVG29-bMSNs@Ce-1F12 recognizes both soluble Aβ_42_ and insoluble Aβ plaques with high binding affinity.

### RVG29-bMSNs@Ce-1F12 inhibits Aβ aggregation

Subsequently, the inhibitory effect of RVG29-bMSNs@Ce-1F12 on Aβ aggregation was evaluated. RVG29-bMSNs@Ce, 1F12, and RVG29-bMSNs@Ce-1F12 were separately mixed with Aβ_42_ and incubated for 48 h at 37 °C to test their performance. Aβ_42_ was mixed with PBS and treated under the same conditions as a control. The level of Aβ_42_ aggregates was detected by sandwich ELISA and ThT. Sandwich ELISA and ThT results showed that the levels of Aβ_42_ aggregation was highest in the PBS group, intermediate in the RVG29-bMSNs@Ce treatment group, and lowest in the RVG29-bMSNs@Ce-1F12 treatment group (Fig. 3d and e). ELISA and ThT data were consistent with the dot blot analysis results, indicating a good efficiency of RVG29-bMSNs@Ce-1F12 in inhibiting Aβ_42_ misfolding (Fig. S9a). On analyzing the morphology of Aβ_42_ in each treatment group, we found that Aβ_42_ mainly existed in the form of β-sheets and typical fibrils in the PBS control group (Fig. 3f(i)). However, in the RVG29-bMSNs@Ce treatment group, Aβ_42_ mainly existed in the form of protofibrils, indicating that RVG29-bMSNs@Ce had a certain inhibitory effect on Aβ_42_ aggregation (Fig. 3f(ii)). In contrast, in the 1F12 (Fig. 3f(iii)) and RVG29-bMSNs@Ce-1F12 (Fig. 3f(iv)) treatment group, only small Aβ_42_ aggregates were observed and the size of the aggregates in the RVG29-bMSNs@Ce-1F12 treatment group was smaller than that in the 1F12 treatment group (Fig. S9b, *p* < 0.0001). These results indicated that RVG29-bMSNs@Ce-1F12 had a good inhibitory effect on Aβ_42_ aggregation.

### RVG29-bMSNs@Ce-1F12 promotes the depolymerization of Aβ fibrils

A dynamic equilibrium exists between the aggregation of monomers and the dissociation of fibrils [43, 44]. Accordingly, we next determined whether the removal of soluble Aβ_42_ by RVG29-bMSNs@Ce-1F12 could accelerate the depolymerization of fibrils. Crude Aβ_42_ fibrils containing monomers and oligomers were dialyzed in PBS buffer containing RVG29-bMSNs@Ce-1F12 (20 µg/mL) to observe the dynamic dissociation of fibrils (Fig. 3g). ThT and ELISA results showed that the level of Aβ_42_ fibrils gradually decreased with an increase in dialysis time (Fig. 3h and i). On analyzing the morphology of Aβ_42_ in different disaggregated states, we found that the morphology of pre-dialysis samples showed β-sheets and typical fibrillar structures and this structure changed into short linear β-sheets and irregular spherical structures after 24 h of dialysis (Fig. 3j). When the dialysis duration was extended to 48 h, more irregular spherical structures were observed, indicating that Aβ fibrils had disaggregated into low-molecular-weight aggregates in the presence of RVG29-bMSNs@Ce-1F12. Thus, RVG29-bMSNs@Ce-1F12 can promote the depolymerization of insoluble Aβ_42_ fibrils.

## RVG29-bMSNs@Ce-1F12 alleviates Aβ aggregates-induced oxidative stress

**Fig. 4:**
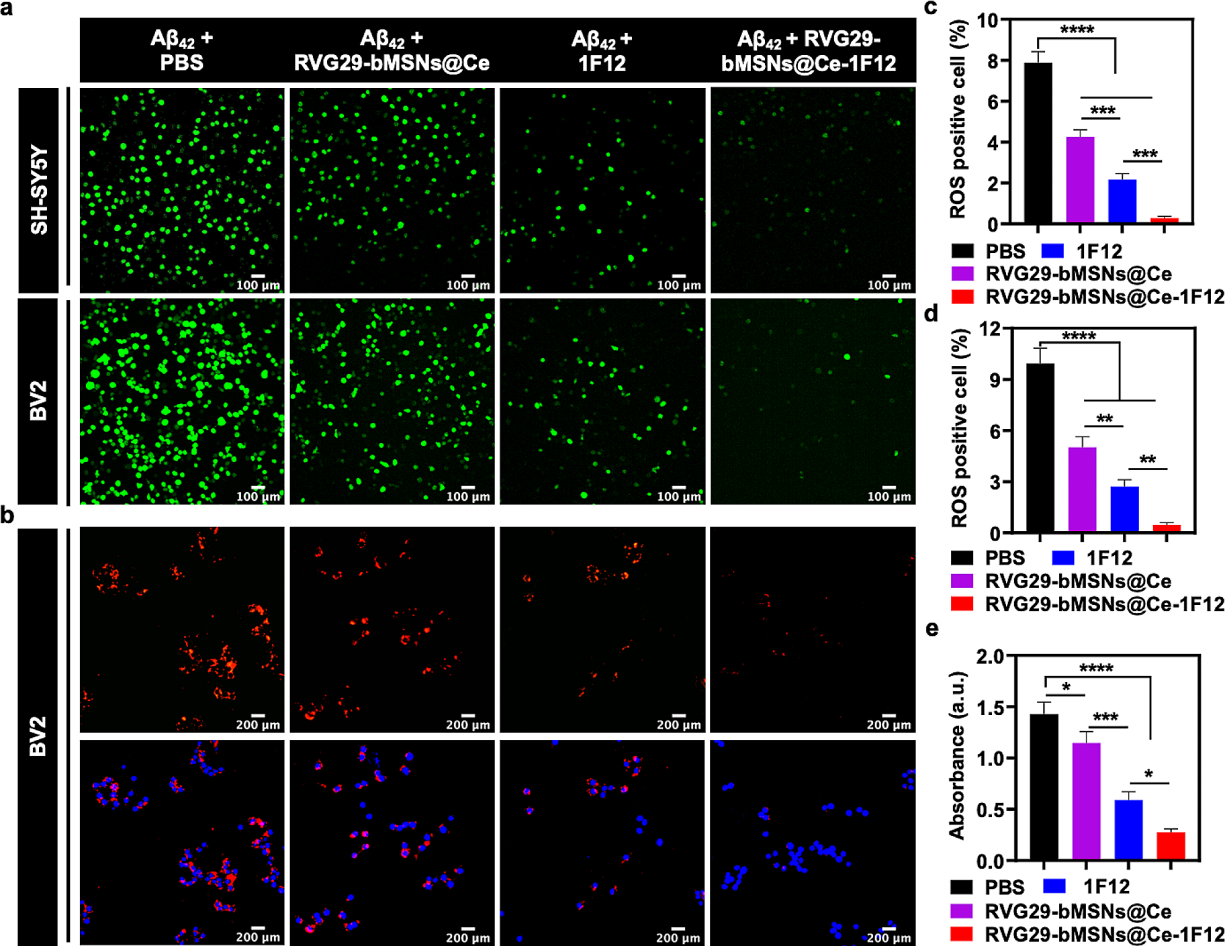
RVG29-bMSNs@Ce-1F12 alleviates Aβ aggregates-induced ROS and microgliosis. DCFH-DA fluorescence (a) and ROS-positive cell area in SH-SY5Y (c) and BV2 cells (d) after exposure to Aβ_42_ in the presence of PBS, RVG29-bMSNs@Ce, 1F12, and RVG29-bMSNs@Ce. Scale bar = 100 μm. Confocal fluorescence image (b) and quantitative analysis (e) of Aβ_42_ aggregates cellular uptake by BV2 cells in each treatment group. Scale bar = 200 μm. Data are presented as means ± SD, *n* = 3. ANOVA was performed for multigroup comparisons, *p < 0.05, **p < 0.01, ***p < 0.001, and ****p < 0.0001

Misfolded Aβ_42_ aggregates have been considered to be a potent mitochondrial toxicant that directly leads to mitochondrial dysfunction and neuronal apoptosis in AD [45, 46]. While evaluating the performance of RVG29-bMSNs@Ce-1F12 in reducing Aβ_42_ aggregate-induced ROS production, DCFH-DA was used as a ROS indicator to examine ROS levels in SH-SY5Y and BV2 cells treated with PBS, RVG29-bMSNs@Ce, 1F12, and RVG29-bMSNs@Ce-1F12. As shown in Fig. 4a, c, and d, a high level of ROS was observed in PBS-treated SH-SY5Y and BV2 cells, indicating Aβ_42_ aggregates can significantly induce the production of ROS. However, in the RVG29-bMSNs@Ce-1F12 treatment group, the weakest ROS fluorescence signal was observed (Fig. 4a, c, and d) due to the potent ROS-scavenging ability of RVG29-bMSNs@Ce (Fig. 2g) and the effective inhibition of Aβ_42_ aggregation by 1F12 (Fig. 3d-f).

Studies have shown that high levels of ROS can effectively induce the proliferation of microglial cells and promote their phagocytosis [47, 48]. As shown in Fig. 4b and e, the PBS group with the strongest ROS fluorescence signal had the highest Aβ_42_ levels. Interestingly, the RVG29-bMSNs@Ce-1F12 treatment group with the weakest ROS fluorescence signal showed the lowest Aβ_42_ levels (Fig. 4b and e). Meanwhile, the morphology of BV2 cells in the PBS-treated group with high oligomer and protofibril levels changed from a short and compact state to an extended and elongated state, resulting in decreased cell viability; however, RVG29-bMSNs@Ce-1F12 treatment reduced the proportion of elongated cells and increased cell viability (Fig. S10). Taken together, these results demonstrated the excellent ability of RVG29-bMSNs@Ce-1F12 in reducing Aβ_42_ aggregate-induced ROS production and microgliosis via potent inhibition of Aβ_42_ aggregation and robust ROS scavenging.

### In vitro and in vivo BBB permeability studies

**Fig. 5:**
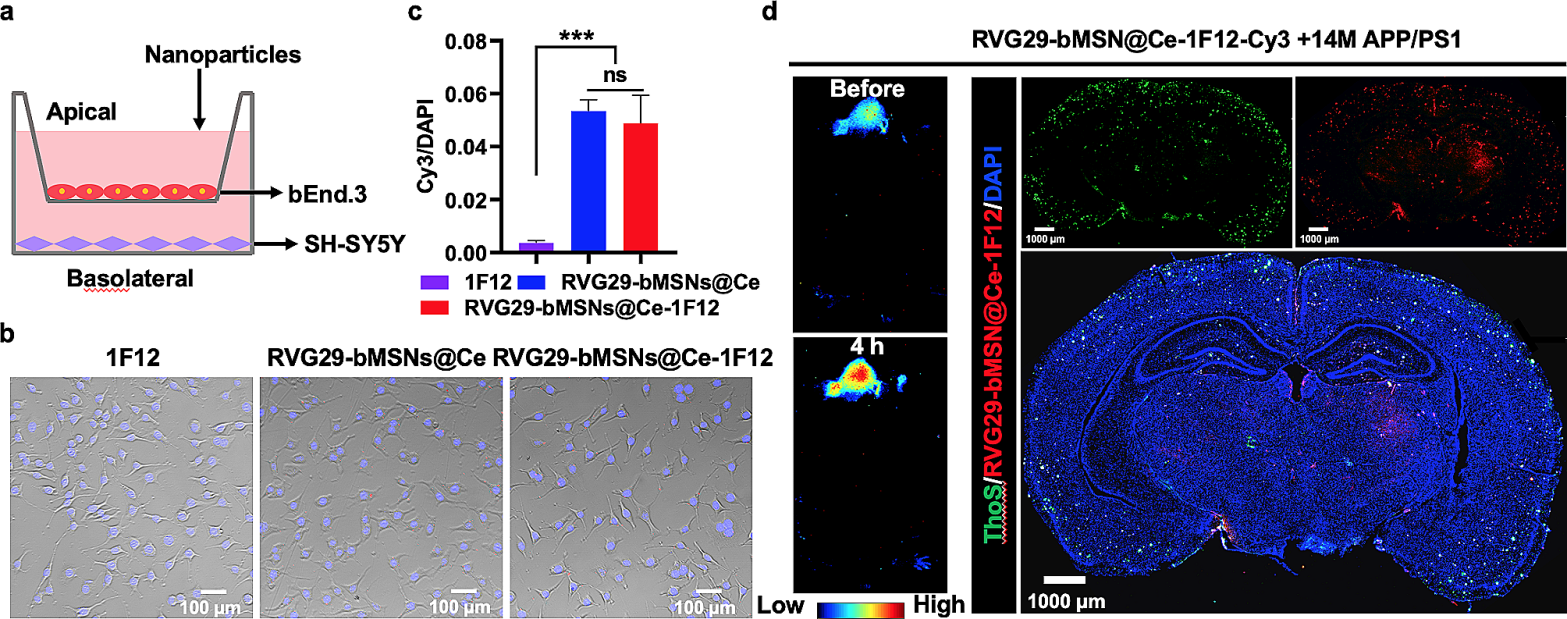
BBB permeability of RVG29-bMSNs@Ce-1F12 in vitro and in vivo. (a) Schematic diagram of in vitro BBB penetration test. Fluorescence images (b) and statistical results (c) of the SH-SY5Y in the basolateral chamber after 4 h incubation with Cy3-1F12, Cy3-RVG29-bMSNs@Ce, and Cy3-RVG29-bMSNs@Ce-1F12. Scale bar = 100 μm. (d) Whole-body fluorescence imaging and confocal fluorescence images of brain Aβ plaques following intravenous administration of RVG29-bMSNs@Ce-1F12. Scale bar = 1000 μm. Data are presented as means ± SD, *n* = 3. ANOVA was performed for multigroup comparisons, *p < 0.05, **p < 0.01, ***p < 0.001, and ****p < 0.0001

RVG29-mediated BBB permeability was first studied in vitro using the endothelial bEnd.3 cell model. Cy3-labeled 1F12, RVG29-bMSNs@Ce, and RVG29-bMSNs@Ce-1F12 were incubated with compact monolayers of bEnd.3 cells grown on transwell inserts, and fluorescence imaging of SH-SY5Y cultured in the bottom chamber were performed (Fig. 5a). The results showed that fluorescent signals were observed in the RVG29-bMSNs@Ce- and RVG29-bMSNs@Ce-1F12-treated groups, but not in the 1F12-treated group, indicating that the antibody could not penetrate the dense monolayer of bEnd. 3 cells (Fig. 5b). In contrast, RVG29-modified nanoparticles showed strong fluorescence signals, and no significant difference was observed between RVG29-bMSNs@Ce- and RVG29-bMSNs@Ce-1F12-treated groups (Fig. 5b and c).

**Fig. 6:**
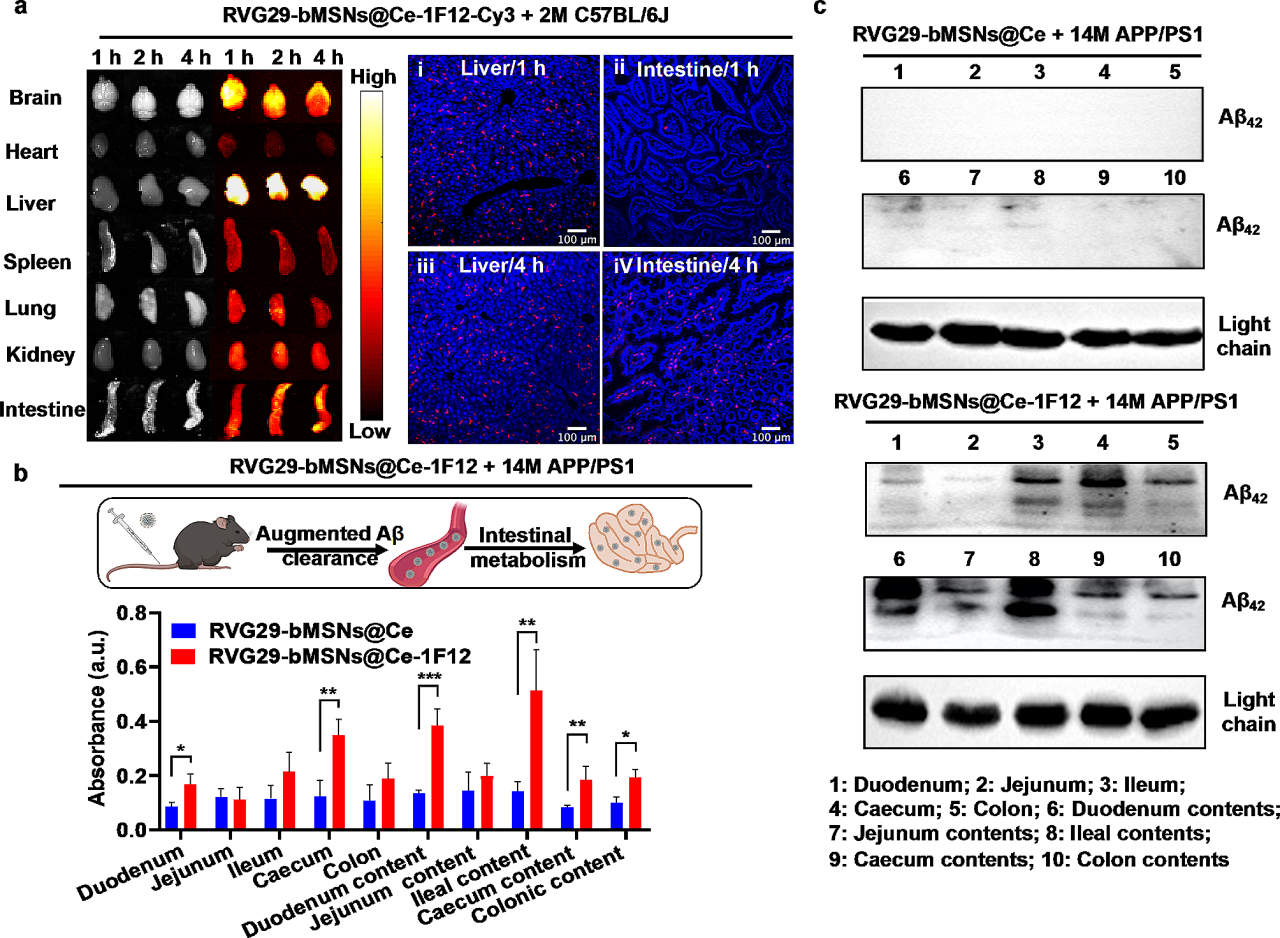
Metabolism of RVG29-bMSNs@Ce-1F12 in vivo. (a) Whole-body fluorescence imaging and confocal fluorescence images of the liver and intestine after intravenous administration. Aβ_42_ levels (b) and isoforms (c) in the intestine of APP/PS1 mice after intravenous administration of RVG29-bMSNs@Ce-1F12 and RVG29-bMSNs@Ce, *n* = 3 per group. ANOVA was performed for multigroup comparisons, *p < 0.05, **p < 0.01, and ***p < 0.001

Furthermore, in vivo BBB permeability studies found that strong fluorescent signals were observed in the brains of C57BL/6J mice after administration of RVG29-bMSNs@Ce and RVG29-bMSNs@Ce-1F12, but not after administration of 1F12, indicating that RVG29 has good BBB permeability (Fig. 6a and Fig. S11). Meanwhile, strong fluorescent signals are also observed in the brains of 14-month-old APP/PS1 mice 4 h after the intravenous injection with RVG29-bMSNs@Ce-1F12 (Fig. 5d). Confocal fluorescence images of whole brain slices showed that brain Aβ plaques were stained by RVG29-bMSNs@Ce-1F12 and co-localized with thioflavin S, indicating that RVG29-bMSNs@Ce-1F12 can cross the BBB and specifically recognize Aβ plaque in the brain (Fig. 5d). Altogether, the results strongly suggest that the RVG29-bMSNs@Ce-1F12 has good BBB permeability.

### Evaluation of the metabolism of RVG29-bMSNs@Ce-1F12 and the pathway of the Aβ_42_ clearance

To evaluate the metabolism of RVG29-bMSNs@Ce-1F12, Cy3-labeled RVG29-bMSNs@Ce-1F12 was intravenously administered to C57BL/6J mice. Cy3-labeled 1F12 and RVG29-bMSNs@Ce were used as controls. The biodistributions of the three fluorescent probes are shown in Fig. 6a and Fig. S11. The results showed that 1F12, bMSNs@Ce-RVG29, and RVG29-bMSNs@Ce-1F12 had similar metabolism pathways from the liver to the intestine. To further explore the fate of Aβ_42_ cleared from peripheral blood, RVG29-bMSNs@Ce-1F12 was intravenously administered to 14-month-old APP/PS1 mice. RVG29-bMSNs@Ce was used as a control. After administration, the duodenum, jejunum, ileum, colon, caecum, and their contents were collected to prepare a homogenate, and Aβ_42_ was then detected in these tissues by sandwich ELISA and IP-Western blot. ELISA results showed that Aβ_42_ levels in intestinal tissues of APP/PS1 mice administered with RVG29-bMSNs@Ce-1F12 were significantly higher than those in the tissues of mice administered with RVG29-bMSNs@Ce, indicating that free Aβ_42_ in the blood could be captured by RVG29-bMSNs@Ce-1F12 and excreted it through intestinal metabolism (Fig. 6b). IP-Western blot image showed that Aβ_42_ monomers and oligomers were detected in the intestine of RVG29-bMSNs@Ce-1F12 treatment group and the level of Aβ_42_ isoforms was significantly higher than those treated with RVG29-bMSNs@Ce (Fig. 6c). Overall, these results demonstrate that RVG29-bMSNs@Ce-1F12 can capture peripheral soluble Aβ_42_ species and excrete them through intestinal metabolism.

### RVG29-bMSNs@Ce-1F12 reduces Aβ levels in the blood and brain in APP/PS1 mice

**Fig. 7:**
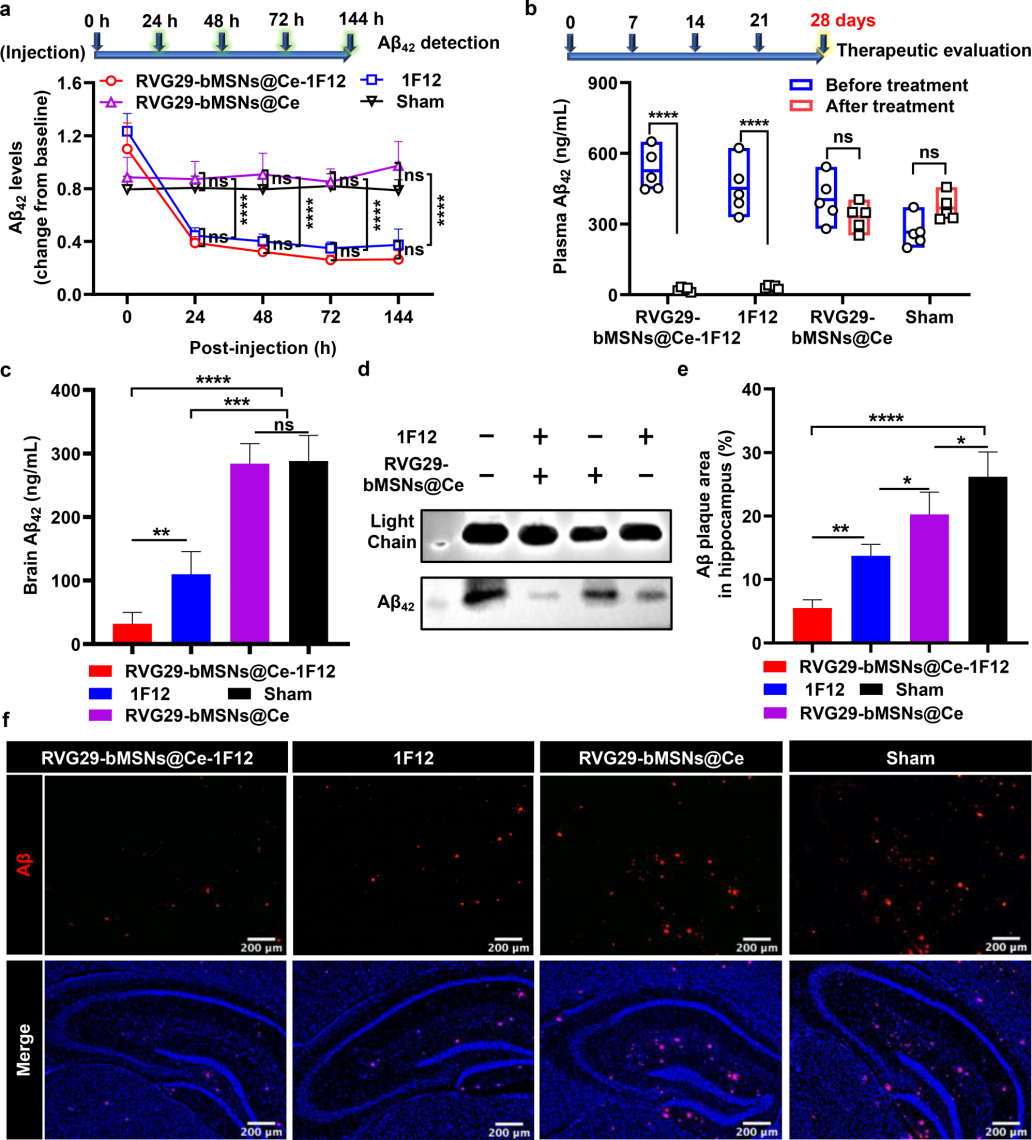
RVG29-bMSNs@Ce-1F12 reduces soluble and insoluble Aβ burden. (a) Dynamic changes in plasma soluble Aβ_42_ levels within 144 h after administration of saline, RVG29-bMSNs@Ce, 1F12, and RVG29-bMSNs@Ce-1F12. Soluble Aβ_42_ levels in the plasma and brain were detected by sandwich ELISA (b, c) and IP-western blot (d) following corresponding treatments. Confocal fluorescence images of Aβ plaques (f) and Aβ plaque-positive fluorescent areas (e) in the hippocampus of APP/PS1 mice after treatments. Scale bar = 200 μm. Data are presented as means ± SD, *n* = 5. ANOVA was used for multigroup comparisons. *p < 0.05, **p < 0.01, ***p < 0.001, and ****p < 0.0001

To evaluate the effect of RVG29-bMSNs@Ce-1F12 on the clearance of Aβ_42_ from peripheral blood, RVG29-bMSNs@Ce-1F12 and RVG29-bMSNs@Ce (10 mg/kg/week), and 1F12 (0.3 mg/kg/week, equal to RVG29-bMSNs@Ce-1F12 antibody amount at injection dose of 10 mg/kg) were separately administered intravenously to 14-month-old APP/PS1 mice for four weeks. Physiological saline was used in the sham control. Mouse plasma was collected and subjected to sandwich ELISA and IP-western blot to assess changes in Aβ_42_ levels. Dynamic monitoring of changes in peripheral Aβ_42_ levels after the administration showed a sharp decrease in Aβ_42_ levels in the RVG29-bMSNs@Ce-1F12 and 1F12 treatment groups, but negligible differences were observed in the RVG29-bMSNs@Ce treatment and sham groups (Fig. 7a). After four weeks of continuous treatment, Aβ_42_ was barely detectable in the RVG29-bMSNs@Ce-1F12 and 1F12 treatment groups, but high levels of soluble Aβ_42_ were observed in the sham and RVG29-bMSNs@Ce treatment groups (Fig. S12). Furthermore, because of the lack of Aβ_42_ targeting, there was no significant difference in plasma Aβ_42_ levels between the sham group and the RVG29-bMSNs@Ce treatment group before and after treatment (Fig. 7b). The abovementioned results indicated that RVG29-bMSNs@Ce-1F12 and 1F12 can rapidly clear Aβ_42_ from the peripheral blood.

We further evaluated the treatment effect of RVG29-bMSNs@Ce-1F12 in the brain. After treatment for four consecutive weeks, the brain tissues of APP/PS1 mice were collected and soluble Aβ_42_ levels were assessed. ELISA results showed that the level of soluble Aβ_42_ in the brains of mice treated with RVG29-bMSNs@Ce-1F12 and 1F12 was significantly lower than that in the brains of mice treated with RVG29-bMSNs@Ce or saline, but the rate of Aβ_42_ clearance by RVG29-bMSNs@Ce-1F12 was significantly higher than that by 1F12 and RVG29-bMSNs@Ce (Fig. 7c). In contrast, brain soluble Aβ_42_ levels showed negligible differences between the sham and RVG29-bMSNs@Ce treatment groups (Fig. 7c). IP-Western blot results were completely consistent with the sandwich ELISA results, indicating that RVG29-bMSNs@Ce-1F12 could significantly reduce the soluble Aβ_42_ levels (Fig. 7d). Fluorescence images of coronal slices of APP/PS1 mouse brain showed that the Aβ plaque load in the hippocampus of RVG29-bMSNs@Ce-1F12-treated mice was significantly lower than that in 1F12- and RVG29-bMSNs@Ce-treated mice, indicating that RVG29-bMSNs@Ce-1F12 effectively inhibited Aβ_42_ aggregation and accelerated the clearance of Aβ_42_ deposits in the brain (Fig. 7e and f).

#### RVG29-bMSNs@Ce-1F12 eliminates excessive ROS and inhibits tau hyperphosphorylation in APP/PS1 mice

**Fig. 8:**
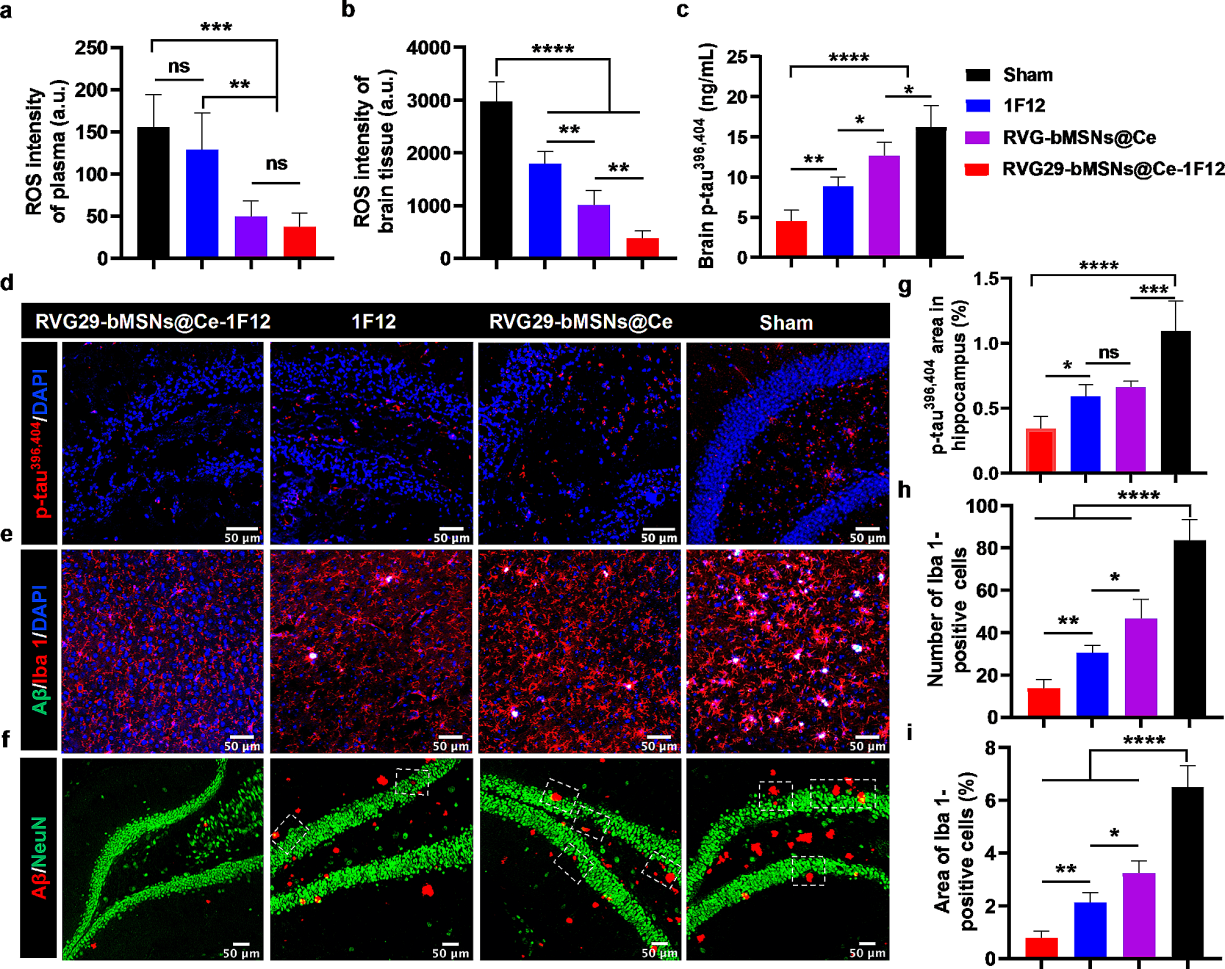
RVG29-bMSNs@Ce-1F12 eliminates excessive ROS and restrains multiple core pathologies. Comparison of ROS levels in the plasma (a) and brain tissue (b) of mice in each treatment groups. (c) Soluble p-tau^396,404^ levels in the brains of mice treated with RVG29-bMSNs@Ce-1F12, 1F12, RVG29-bMSNs@Ce, and saline. Fluorescence images (d) and area (g) of p-tau^396,404^ aggregates in the hippocampus of APP/PS1 mice after treatments. Fluorescence images (e), number (h), and area (i) of Iba 1-positive cells in the brain tissues of APP/PS1 mice after corresponding treatments. (f) Fluorescence images of NeuN in the hippocampus of APP/PS1 mice after treatments. Scale bar = 50 μm. Data are presented as means ± SD, *n* = 5. ANOVA was performed for multigroup comparisons. *p < 0.05, **p < 0.01, ***p < 0.001, and ****p < 0.0001

As the core pathogenesis of AD, oxidative stress is considered to be a key “bridge” connecting various pathogenesis and pathways of AD [49, 50]. In vitro test results indicated that RVG29-bMSNs@Ce-1F12 had strong ROS scavenging ability. Therefore, we evaluated ROS levels in peripheral blood and brain tissue for each treatment group. Figure 8a shows that the levels of ROS in the plasma of mice in the sham and 1F12 treatment groups were higher than those of mice in the RVG29-bMSNs@Ce and RVG29-bMSNs@Ce-1F12 treatment groups. Similarly, this phenomenon was also observed in brain tissue, but the ROS elimination effect of the RVG29-bMSNs@Ce-1F12 treatment group was more significant than that of the RVG29-bMSNs@Ce and 1F12 groups (Fig. 8b). This may be due to the coordinated inhibition of Aβ_42_ aggregation by 1F12, thereby reducing the production of ROS during the aggregation process (Fig. 4a, c, and d) and the strong ROS scavenging ability of bMSNs@Ce (Fig. 2g).

Mounting evidence supported that the Aβ_42_ aggregates and excessive ROS can induce tau hyperphosphorylation and hyperphosphorylated tau aggregation [11, 51, 52]. Tau phosphorylation at Ser396 and Ser404 (p-tau^396,404^) is one of the earliest events in AD and p-tau^396,404^ is the main component of NFTs [53–55]. Inhibition of tau hyperphosphorylation and hyperphosphorylated tau aggregation is an important aspect of AD treatment. Dot blot and ELISA using specific anti-p-tau^396,404^ antibodies showed high levels of soluble p-tau^396,404^ in the sham group [37, 56], whereas treatment with RVG29-bMSNs@Ce and 1F12 resulted in decreased p-tau^396,404^ levels (Fig. 8c and Fig. S13). Notably, the lowest levels of p-tau^396,404^ were observed in the RVG29-bMSNs@Ce-1F12 group (Fig. 8c and Fig. S13). In addition, fluorescence images of insoluble p-tau^396,404^ in the brains of APP/PS1 mice revealed extensive p-tau^396,404^ aggregates in the hippocampus of mice in the sham group (Fig. 8d and g). In contrast, the fluorescent signal of p-tau^396,404^ aggregates were significantly reduced in the brains of APP/PS1 mice treated with RVG29-bMSNs@Ce or 1F12. However, compared with RVG29-bMSNs@Ce and 1F12 treatment groups, RVG29-bMSNs@Ce-1F12 treatment group showed lower levels of p-tau^396,404^ aggregates (Fig. 8d and g). These results suggested that both intravenous administration of 1F12 and RVG29-bMSNs@Ce can reduce the levels of Aβ_42_ and p-tau^396,404^ in the brains of APP/PS1 mice via different mechanisms. 1F12 reduces brain Aβ load mainly by inhibiting Aβ_42_ misfolding and attenuating Aβ-induced tau phosphorylation [57, 58]. In contrast, RVG29-bMSNs@Ce can eliminate excessive ROS, thereby reducing oxidative stress damage and inhibiting hyperphosphorylated tau or Aβ aggregation. Thus, RVG29-bMSNs@Ce-1F12 treatment enhanced the therapeutic effect by combining the advantages of both 1F12 and RVG29-bMSNs@Ce.

#### RVG29-bMSNs@Ce-1F12 attenuates microgliosis in APP/PS1 mice

Aβ and ROS in AD brains are neurotoxic and directly activate microglia, leading to neuroinflammation [4, 48, 59]. The ability of RVG29-bMSNs@Ce-1F12 to attenuate microgliosis in vivo was evaluated. In the sham group, immunofluorescence staining of brain sections with ionized calcium-binding adaptor molecule-1 (Iba 1, a biomarker of microglia) showed a strong signal for Iba 1-positive cells, indicating that the microglia in the 14-month-old APP/PS1 mice had been activated (Fig. 8e). Compared with the sham group, the RVG29-bMSNs@Ce-1F12, 1F12, and RVG29-bMSNs@Ce treatment groups showed significantly reduced Iba 1-positive signal in the mouse brain tissues; the number and area of Iba 1-positive cells in the RVG29-bMSNs@Ce-1F12 treatment group were the lowest, indicating that RVG29-bMSNs@Ce-1F12 effectively attenuates microgliosis in vivo (Fig. 8h and i).

**Fig. 9:**
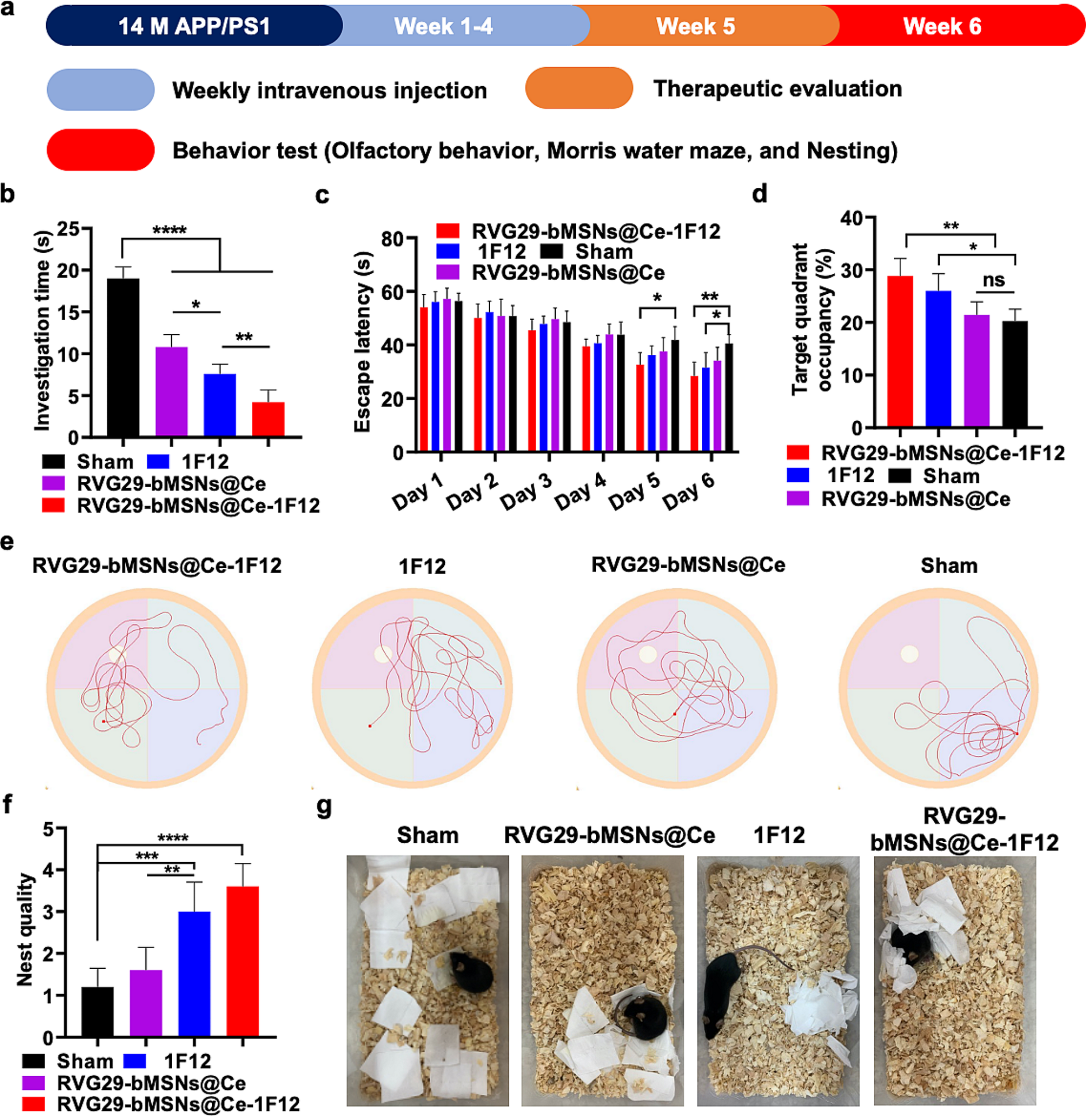
RVG29-bMSNs@Ce-1F12 ameliorates cognitive and olfactory dysfunction in APP/PS1 mice. (a) Schedule of treatment and therapeutic evaluation. (b) Investigation times for APP/PS1 mice in different treatment groups. Escape latency from the hidden platform (c), percentage of dwell time (d), and representative path tracing (e) in the platform quadrant for each treatment group. Nest quality (f) and representative images (g) of APP/PS1 mice in different treatment groups. Data are presented as means ± SD, *n* = 5. *p < 0.05, **p < 0.01, and ****p < 0.0001

## RVG29-bMSNs@Ce-1F12 ameliorates olfactory and cognitive dysfunction in APP/PS1 mice

Figure 8f shows the neuronal loss in dentate gyrus region, especially in Aβ plaque enriched region, but the loss of neurons was significantly improved in the RVG29-bMSNs@Ce-1F12 treatment group with low levels of Aβ plaques and ROS. It is well-known that along with the massive loss of neurons, olfactory dysfunction, memory loss, and cognitive dysfunction are typical clinical manifestations of AD [60, 61].To evaluate the therapeutic effect of RVG29-bMSNs@Ce-1F12 for improving olfactory and cognitive dysfunction in 14-month-old APP/PS1 mice, a series of tests were conducted including olfactory behavior analysis, Morris water maze testing, and nest-building assessments (Fig. 9a). As shown in Fig. 9b, APP/PS1 mice in the RVG29-bMSNs@Ce-1F12 treatment group took lesser time to smell the target (∼ 4.2 s), followed by the mice in 1F12 (∼ 7.6 s) and RVG29-bMSNs@Ce (∼ 12.2 s) treatment groups, whereas the mice in the sham group took more than 18 s to smell the target, and most of the mice in the sham group failed to smell the target, indicating that RVG29-bMSNs@Ce-1F12 improved olfactory impairments.

In the Morris water maze test, the APP/PS1 mice in the sham group spent longer searching for the platform during the hidden platform stage of the tests, showing severely impaired learning and spatial memory functions (Fig. 9c). After treatment with RVG29-bMSNs@Ce-1F12, the escape latency was significantly reduced and was better than that observed in the 1F12 and RVG29-bMSNs@Ce treatment group mice (Fig. 9c). Furthermore, there was no significant difference in swimming speed among all groups, indicating that the locomotor behavior and vision of mice were not affected after treatment with RVG29-bMSNs@Ce or 1F12 (Fig. S14a). In the stage of spatial exploration after removing the hidden platform, the mice in the sham group stayed in the platform quadrant for a shorter time than those in the RVG29-bMSNs@Ce-1F12 and 1F12 treatment groups, and their movement paths were random and disordered, indicating that these mice had severe memory dysfunction (Fig. 9d and e). Of note, RVG29-bMSNs@Ce-1F12-treated mice exhibited a spatially oriented swimming path. They spent the longest time in the platform quadrant (Fig. 9d and e) and crossed the platform the greatest number of times (Fig. S14b), which indicates that RVG29-bMSNs@Ce-1F12 significantly reduced memory impairment in APP/PS1 mice.

The nest of APP/PS1 mice in the sham group was disorganized (Fig. 9f and g), indicating cognitive deficits. In contrast, the nest quality of mice in the RVG29-bMSNs@Ce-1F12 treatment group was the highest (superior to that of mice in the 1F12 and RVG29-bMSNs@Ce treatment groups) (Fig. 9f and g). Taken together, our findings demonstrated that RVG29-bMSNs@Ce-1F12 could improve cognitive and olfactory dysfunction in APP/PS1 mice.

## Conclusion

In summary, we designed and constructed a dual-targeted multifunctional nanocomposite, RVG29-bMSNs@Ce-1F12, for combination therapy of AD. Brain-targeting RVG29 peptide and anti-Aβ_42_ antibody 1F12 were immobilized on the surface of bMSNs to capture peripheral and CNS Aβ_42_ and inhibit Aβ_42_ oligomerization in the brain. In addition, loading CeNPs on the surface of RVG29-bMSNs@Ce-1F12 helped effectively scavenge excess ROS, thereby alleviating oxidative stress, attenuating microgliosis, and restraining hyperphosphorylated tau and Aβ aggregation. Compared with 1F12 and RVG29-bMSNs@Ce alone, the multifunctional RVG29-bMSNs@Ce-1F12 nanocomposite achieved a synergistic therapeutic effect. The encouraging results observed with RVG29-bMSNs@Ce-1F12 treatment in APP/PS1 mice indicated that Aβ_42_ and ROS dual-targeted passive immunotherapy could significantly reduce multiple core pathological burdens and ameliorate cognitive impairment. This study provides insight into the design of dual-targeted multifunctional nanoplatforms with more focus on the inhibition of Aβ_42_ aggregation and scavenging of ROS for AD therapy.

### Electronic supplementary material

Below is the link to the electronic supplementary material.

Supplementary Material 1

## Data Availability

No datasets were generated or analysed during the current study.

## Declarations

### Supporting information

Synthetic scheme of protein-modified bMSNs@Ce (Fig. S1); The size and Zeta potential of Nanoparticles (Fig. S2); XPS analysis of RVG29-bMSNs@Ce-1F12 (Fig. S3); The AFM image of RVG29-bMSNs@Ce-1F12 (Fig. S4); Characterization of RVG29-bMSNs@Ce-1F12 (Fig. S5); Hydroxyl radical scavenging test (Fig. S6); Biocompatibility assay (Fig. S7); Characterization of Aβ plaque stained with 1F12 and RVG29-bMSNs@Ce (Fig. S8); Evaluation of the ability of RVG29-bMSNs@Ce-1F12 to inhibit Aβ aggregation (Fig. S9); RVG29-bMSNs@Ce-1F12 alleviates Aβ_42_ aggregates-induced microgliosis (Fig. S10); Biodistribution of 1F12 and RVG29-bMSNs@Ce (Fig. S11); IP-Western blot analysis of Aβ_42_ level in plasma of APP/PS1 mice after different treatments (Fig. S12); Soluble p-tau^396,404^ levels in the brains of mice treated with RVG29-bMSNs@Ce-1F12, 1F12, RVG29-bMSNs@Ce, and saline were detected by dot blot (Fig. S13); RVG29-bMSNs@Ce-1F12 ameliorates learning and memory impairments in APP/PS1 mice (Fig. S14).
