# Supplementary material for: Aβ42 and ROS dual-targeted multifunctional nanocomposite for combination therapy of Alzheimer’s disease
Source: J Nanobiotechnology. 2024 May 23;22:278. doi: 10.1186/s12951-024-02543-z (PMC11112798; doi:10.1186/s12951-024-02543-z)
Supplement: Supplementary file 1 — Supplementary Material 1 [file 12951_2024_2543_MOESM1_ESM.docx]

- Supplementary Information-

**Aβ_42_ and ROS Dual-targeted Multifunctional Nanocomposite for Combination Therapy of Alzheimer’s Disease**

Liding Zhang^1,2†^, Kai Cao^2†^, Jun Xie^2^, Xiaohan Liang^2^, Hui Gong^2,3^, Qingming Luo^1,3*^, Haiming Luo^1,2,3*^

^1^State Key Laboratory of Digital Medical Engineering, Key Laboratory of Biomedical Engineering of Hainan Province, School of Biomedical Engineering, Hainan University, Haikou 570228, China

^2^Britton Chance Center for Biomedical Photonics, Wuhan National Laboratory for Optoelectronics, MoE Key Laboratory for Biomedical Photonics, Huazhong University of Science and Technology, Wuhan 430074, China

^3^HUST-Suzhou Institute for Brainsmatics, JITRI, Suzhou 215123, China

^†^These authors contributed equally to this work.

*Corresponding author: Haiming Luo, [hemluo@hust.edu.cn](mailto:hemluo@hust.edu.cn); Qingming Luo, qluo@hainanu.edu.cn

Key Laboratory of Biomedical Engineering of Hainan Province, School of Biomedical Engineering, Hainan University, 570228, Haikou, China;

**Supplementary Figures**


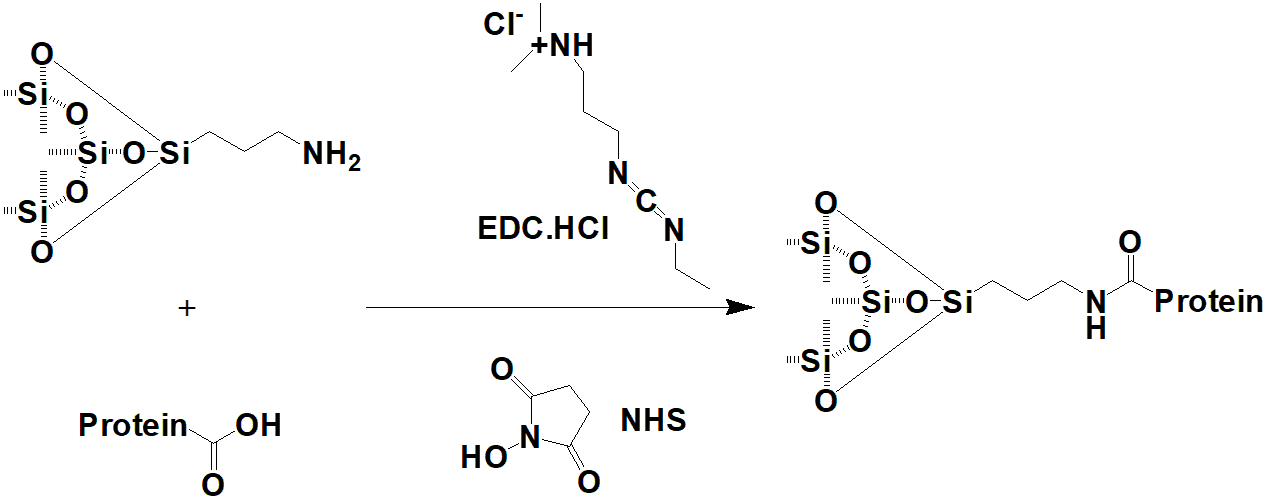


**Fig. S1** Synthetic scheme of protein-modified bMSNs@Ce.

**Fig. S2** The size and Zeta potential of Nanoparticles. Hydrodynamic diameters of bMSNs (a), CeNP (b), and bMSNs@Ce (c). (d) The zeta potential data of bMSNs, bMSNs@Ce, and RVG29-bMSNs@Ce-1F12. Data are presented as means ± SD, n = 3.

**Fig. S3** X-ray photoelectron spectroscopy (XPS) analysis of RVG29-bMSNs@Ce-1F12.

**Fig. S4** The atomic force microscope image (a) and reconstruction of the three walls (b) of RVG29-bMSNs@Ce-1F12.

**Fig. S5** Characterization of RVG29-bMSNs@Ce-1F12. The RVG29 and 1F12 on the surface of nanoparticles were identified by ELISA (a) and SDS-PAGE (b). Data are presented as means ± SD, n = 3. **p* < 0.05 and ***p* < 0.01.

Fig. S6 Neutralization of hydroxyl radical by RVG29-bMSNs@Ce-1F12 in a dose-dependent manner. Data are presented as mean ± SD, n = 3.

**Fig. S7** Biocompatibility assay. (a) The morphology of SH-SY5Y and BV2 cells before and after incubation with RVG29-bMSNs@Ce-1F12. Scale bar = 100 μm. (b) H&E staining of the main organs after RVG29-bMSNs@Ce-1F12 treatment. (c) The color change of hemolysis in supernatant of red blood cells after treated with different concentrations of RVG29-bMSNs@Ce-1F12 (0–3.2mg/mL). Scale bar = 100 μm.

**Fig. S8** Characterization of Aβ plaque stained with 1F12 and RVG29-bMSNs@Ce. Confocal fluorescence images of Aβ plaque stained with 1F12 (a) and RVG29-bMSNs@Ce (b). Thioflavin S (green) was used as the positive control. Scale bar = 100 μm.

**Fig. S9** Evaluation of the ability of RVG29-bMSNs@Ce-1F12 to inhibit Aβ aggregation. (a) Dot blot analysis of Aβ_42_O levels in the treatment groups of PBS, RVG29-bMSNs, 1F12, and RVG29-bMSNs@Ce-1F12. (b) Comparison of the particle size of Aβ_42_ aggregates in the 1F12 and RVG29-bMSNs@Ce-1F12 treatment groups.

**Fig. S10** RVG29-bMSNs@Ce-1F12 alleviates Aβ_42_ aggregates-induced oxidative stress and microgliosis. Morphological changes (a), percentage of long cells (b), and cell viability (c) after exposure to Aβ_42_ in the presence of PBS and RVG29-bMSNs@Ce-1F12. Scale bar = 100 μm. Data are presented as means ± SD, n = 3. ANOVA was performed for multigroup comparisons, ***p* < 0.01, ****p* < 0.001, and *****p* < 0.0001.

**Fig. S11** The tissue distribution of 1F12 and RVG29-bMSNs@Ce. Time course fluorescence imaging of organs from C57BL/6J mice after administered with Cy3-labeled 1F12 and RVG29-bMSNs@Ce.

**Fig. S12** IP-Western blot analysis of Aβ_42_ level in plasma of APP/PS1 mice after treated with normal saline, RVG29-bMSNs@Ce, 1F12, and RVG29-bMSNs@Ce-1F12.

**Fig. S13** Soluble p-tau^396,404^ levels in the brains of mice treated with RVG29-bMSNs@Ce-1F12, 1F12, RVG29-bMSNs@Ce, and saline were detected by dot blot.

**Fig. S14** RVG29-bMSNs@Ce-1F12 ameliorates learning and memory impairments in APP/PS1 mice. (a) Swimming speed of APP/PS1 mice in the hidden platform learning trials and probe trial. (b) The platform crossing times of APP/PS1 mice in probe trial after treatments. Data are presented as means ± SD, n = 5. **p* < 0.05 and ***p* < 0.01.
